# Patient Perspectives on Accessing Acute Illness Care

**DOI:** 10.5811/westjem.2017.3.33289

**Published:** 2017-05-15

**Authors:** Mary K. Finta, Amy Borkenhagen, Nicole E. Werner, Joyce Duckles, Craig R. Sellers, Sandhya Seshadri, Denise Lampo, Manish N. Shah

**Affiliations:** * University of Wisconsin Madison, School of Medicine and Public Health, Berbee Walsh Department of Emergency Medicine, Madison, Wisconsin; † University of Wisconsin Madison, College of Engineering, Department of Industrial and Systems Engineering, Madison, Wisconsin; ‡ University of Rochester, Warner School of Education and Human Development, Rochester, New York; § University of Rochester, School of Nursing, Rochester, New York

## Abstract

**Introduction:**

Older adults use the emergency department (ED) at high rates, including for illnesses that could be managed by their primary care providers (PCP). Policymakers have implemented barriers and incentives, often financial, to try to modify use patterns but with limited success. This study aims to understand the factors that influence older adults’ decision to obtain acute illness care from the ED rather than from their PCPs.

**Methods:**

We performed a qualitative study using a directed content analysis approach from February to October 2013. Fifteen community-dwelling older adults age≥65 years who presented to the ED of an academic medical center hospital for care and who were discharged home were enrolled. Semi-structured interviews were conducted initially in the ED and subsequently in patients’ homes over the following six weeks. All interviews were audio-recorded, transcribed, verified, and coded. The study team jointly analyzed the data and identified themes that emerged from the interviews.

**Results:**

The average age of study participants was 74 years (standard deviation ±7.2 years); 53% were female; 80% were white. We found five themes that influenced participants’ decisions to obtain acute illness care from the ED: limited availability of PCP-based care, variable interactions with healthcare providers and systems, limited availability of transportation for illness care, desire to avoid burdening friends and family, and previous experiences with illnesses.

**Conclusion:**

Community-dwelling older adults integrate multiple factors when deciding to obtain care from an ED rather than their PCPs. These factors relate to personal and social considerations, practical issues, and individual perceptions based on previous experiences. If these findings are validated in confirmatory studies, policymakers wishing to modify where older adults receive care should consider person-centered interventions at the system and individual level, such as decision support, telemedicine, improved transport services, enhancing PCPs’ capabilities, and enhancing EDs’ resources to care for older patients.

## INTRODUCTION

The 46.2 million older adults (age≥65 years) residing in the United States require medical care frequently for acute illnesses, making over 20 million visits to emergency departments (EDs) annually.^1^ Policymakers have implemented incentives and barriers, often financial, to encourage older patients to obtain acute illness care from their primary care providers (PCP). This work has been driven by a desire to reduce healthcare expenditures, but interventions have had limited success.^2^,^3^,^4^,^5^ More recently, researchers and clinicians have recognized that acute illness care in a PCP’s office may offer advantages over ED-based care. Among the proposed benefits are enhancing continuity of care for complex older patients, avoiding the challenging ED environment with its prevalent infectious illnesses, excessive noise, inadequate lighting, frequent interruptions, and insufficient nourishment, and potentially avoiding the impaired cognition, mood, and functional status often experienced by older adults following ED care for minor problems.^6^,^7^,^8^,^9^,^10^ In contrast, the ED, unlike the PCP’s office, can provide the extensive diagnostic testing and therapeutic interventions needed by older adult patients.^11^,^12^

Patients generally have a limited role in this discussion of the optimal location for acute illness care. Studies have shown that they usually have robust relationships with their PCPs and thus would likely access their PCPs for care.^13^ The Emergency Medicine Patients’ Access to Healthcare (EMPATH) study found that medical necessity, ED preference, convenience, affordability, and insurance limitations were the primary reasons for seeking ED care.^14^ Rust and colleagues also found that practical barriers to accessing the PCP for acute care exist, such as a lack of transportation and of appointments.^15^ Other studies have focused on demographic and clinical factors associated with patients who use the ED. Older adults with certain diagnoses, a hospital admission within the previous six months, a history of alcohol abuse, and poor overall health, among other characteristics, use the ED more frequently than others.^16^,^17^ However, little research has explored in depth how or where older adults obtain care for acute illnesses, and few researchers have specifically examined system-level factors.^18^,^19^ Furthermore, to our knowledge no studies have directly queried older adults to fully understand the factors that influence where they obtain care for their acute illnesses. This hypothesis-generating qualitative study aimed to identify the factors that influence older adults decision to seek care from the ED, rather than their PCPs, for acute illnesses.

## METHODS

This research is one aim of a larger study whose purpose is to broadly examine how community-dwelling older adults manage their care and navigate their healthcare and health concerns within the context of their lives surrounding an ED visit. The larger study aims to uncover, from the patient and caregiver perspectives, the supports and constraints that shape the ED-to-community transitioning process. One specific goal within this work is to build a better understanding of the factors that influence whether they obtain medical care from the ED, rather than their PCPs, for acute illness symptoms. The study was approved with informed consent by the University of Rochester Research Subjects Review Board and the University of Wisconsin-Madison Institutional Review Board. All COREQ criteria were met apart from our inability to retain records of individuals approached who declined inclusion in the study.

**Population Health Research Capsule**

What do we already know about this issue?

*Older adults use the ED at high rates, including for illnesses that could be managed by their primary* *care* *providers (PCP), despite attempts to decrease ED use.*

What was the research question?

*What factors influence older adults’ decision to obtain* *acute* *illness* *care* *from the ED rather than their PCPs?*

What was the major finding of the study?

*Older adults integrate many non-medical factors when deciding to seek* *care* *from an ED instead of their PCPs.*

How does this improve population health?

*For more efficient* *acute* *illness* *care* *for older adults, planners must develop and integrate person-centered* *care* *concepts into a complex healthcare system.*

### Study Setting

This study took place in Rochester, New York. All subjects were identified and consented in the University of Rochester Medical Center ED, which is an academic medical center and Level I trauma center ED that cares for approximately 100,000 patients per year.

### Study Subjects

A convenience sample of community-dwelling older adult ED patients (age≥65 years) was recruited from February 2013 to October 2013 between 9 am and 9 pm when a study investigator was available. Potential subjects were excluded if they lived in skilled nursing facilities or assisted living facilities, lacked decisional capacity, could not communicate in English, presented for alcohol intoxication, or had received care in an ED within the previous 30 days. In addition, patients needed to be discharged from the ED to their homes to be eligible for participation in the study. All participants provided informed consent, along with any caregivers who were present and were willing to be included in the interviews.

### Study Methods

The study team developed a semi-structured interview guide based on the aims of the larger study and the existing literature. The guides were iteratively revised based on study team review and pilot testing. Additionally, we completed chart reviews for basic demographic and clinical information. In the ED, the initial interview explored circumstances that contributed to the ED visit, perceptions of health, social relationships, anticipated challenges upon discharge, and relationships with the participant’s PCP.

After discharge, participants were interviewed in their homes up to two times, approximately two weeks apart, over a six-week period. Interviews were framed as conversations and built upon the data gathered in previous conversations. Participants and caregivers discussed the acute illnesses that led to the ED visit, their reasons for choosing the ED over a visit to the PCP, their perceptions of health and challenges associated with staying healthy, their personal, social and health priorities, and their relationships with medical systems, PCPs, and social support structures. The interview in the ED lasted approximately 30 minutes, and each of the in-home interviews lasted approximately one hour. We collected a total of 728 pages of transcripts.

All interviews (n=36) were audio recorded, transcribed verbatim, and verified. The study team ceased enrollment when data saturation was achieved and no new information was being obtained through the qualitative interviews for any of the aims of the parent study. To evaluate saturation, the team reviewed the transcripts of the interviews after every 2–3 subjects had completed study procedures. When the team agreed no new information was being collected, we decided to cease new enrollment.

### Data Coding and Analysis

This study, which aimed to identify the factors influencing whether older adults elected to seek care from the ED rather than their PCP, was a pre-planned analysis of data gathered for the larger qualitative study. We conducted data analysis using methods consistent with directed content analysis approaches to research and analysis.^20^ Codes were derived based on a synthesis of the literature and previous pilot work.^4^,^8^,^13^,^15^ The study team also identified in-vivo codes within the data. To ensure consistency in coding, six transcripts were coded independently by the team, and then codes were compared and discussed as a group. Two study team members then coded all transcripts (AB, MKF) using NVivo software; 20% of these final coded transcripts were systematically verified for consistency and accuracy by two other team members (MNS, NEW). The percent agreement function in NVivo was used to crosscheck coding between researchers, and any individual codes or transcripts that did not exhibit an agreement of at least 80% were recoded. All transcripts had an agreement of greater than 80%. Study team members then jointly identified themes that emerged from the data.

## RESULTS

For the cohort, the average age was 74 years (standard deviation 7.2 years); seven males and eight females participated; three participants were Black and the remaining participants were White (Table 1). We identified five themes that reflect the factors contributing to whether participants chose to obtain acute illness care from the ED, rather than their PCP, which are detailed below. Of note, biomedical concerns did not emerge as a factor in choosing one site over another; in other words, participants did not describe choosing one site of care over another due to the severity of their illness.

### Theme 1: Limited availability of PCP-based illness care

Some participants commented upon their ability to see their PCPs whenever needed (Table 2, Quote 1–2), while others commented on their inability to obtain care from their PCPs and the convenience of ED-based care (Table 2, Quote 3–5). No comments clearly explained the difference between these two responses. The lack of PCP availability after hours and on weekends was noted by participants; no participant indicated that their PCP was available after hours and on weekends (Table 2, Quote 3–4).

### Theme 2: Variable interactions with healthcare providers and systems

Participants remarked upon their positive and productive working relationships with their PCPs (Table 2, Quote 6–8). Participants provided comments describing positive interactions as they obtained the care they desired (Table 2, Quote 9–10). Participants also noted that PCPs often referred them to the ED because the PCPs could not provide the services needed in the office (Table 2, Quote 11–13). A few participants claimed the ED was preferred (Table 2, Quote 14). No participants identified a conflict in their positive relationships with their PCPs despite using the ED for care.

### Theme 3: Limited availability of transportation for illness care

Participants spoke extensively about the problems they encountered procuring transportation to perform healthcare-related tasks (e.g., physician appointments) because they did not drive (Table 2, Quote 15). Public transport problems included 1) poor availability of transportation; 2) lack of available transportation for emergent appointments; and 3) poor reliability of public transportation providers. When public transport was used, participants described multi-hour trips that were exhausting (Table 2, Quote 16–18). The alternative was to ask friends and family to assist with their needs, which the participants did not want to do (Table 2, Quote 19).

### Theme 4: Desire to avoid burdening friends and family

Participants expressed their fears of burdening friends and family for maintaining health-related appointments. They specifically highlighted their discomfort and dislike with having to trouble family or friends for help with taking them to appointments or with their complex healthcare needs (Table 2, Quote 20–22). This discomfort even extended to non-health related support, such as grocery shopping.

### Theme 5: Previous experiences with illnesses

Participants commented on their experiences with previous acute illnesses, their reactions to those episodes, and how these experiences and reactions influenced their decision-making regarding the site of their care. Some participants discussed their inability to tolerate uncertainty related to their symptoms, with many choosing to present at the ED because, as one patient put it, “if I hadn’t come in, I would have always wondered if I should have.” Others discussed the convenience of obtaining care whenever they needed it (Table 2, Quote 23–27). Some patients discussed their previous experiences, and how those experiences led to accessing ED care (Table 2, Quote 28). Finally, participants remarked that the opinions of their friends and family members weighed heavily on their decision as to where to seek care (Table 2, Quote 29–31).

## DISCUSSION

In this qualitative study, we found that a wide variety of factors influence whether older adults obtain acute illness care from the ED rather than their PCPs. Most of these factors were unrelated to their medical symptoms or to the severity of their illness. Instead, they stemmed from personal and social considerations, practical considerations, and perceptions based on previous experiences. Policymakers who desire to modify the location at which older adults obtain acute illness care must consider interventions, primarily at the systems level, to address these issues.

Care availability proved to be a significant factor that influenced the site for acute illness care. Consistent with the literature, the participants in this study spoke highly of their PCPs and their relationship with them.^21^,^22^ However, they struggled with the system of care in which their PCPs operate: participants highlighted the problems they experienced in obtaining care when they needed it, particularly after hours and during weekends, without going to the ED. They also discussed being referred to an ED to obtain the necessary care, despite their requests to be cared for in the PCPs’ offices. These barriers are not surprising as PCP offices have limited hours and limited ability to perform diagnostic testing and deliver treatments, which are frequently needed by ill older adults.^8^,^11^,^15^,^23^

Participating older adults also described their struggles with acquiring transportation to appointments because those who did not drive wished not to burden friends and family members. For some, an insurance-based transportation system was available, but they lamented its poor reliability and lack of on-demand availability (Table 2, Quote 15–21).

Older adults’ experiences with illness, and their reaction to their illnesses, played a substantial role in where they sought care. Participants noted that their previous experience of being referred to the ED for acute illness care had led to their decision to access ED care. It was also clear that worries about their health and the uncertainty of their conditions, particularly in the setting of multiple comorbidities, drove participants to obtain immediate care in the ED. This finding is consistent with other research studies, which have found that high anxiety related to the implications of illnesses can act as a strong driving force in choosing the ED for its immediacy of care.^18^,^24^

Interventions exist that could address the factors described by community-dwelling older adults as influencing their preferred site for acute illness care. While no single intervention will likely apply to all community-dwelling older adults, an approach that places the individual at the center of the system may have benefit. Westphal describes this need for individualization when he advocates for *person*-centered care.^25^ Considering the notion of person-centered care with the themes from our participants regarding the difficulties they encounter in navigating a complex healthcare system, it is clear that any acute illness care delivery system needs to be flexible for the diversity of patients and their situations, and needs to consider the intensity of healthcare required by these patients.

A number of potential interventions could operate at the system level. A major consideration is where acute illness care is available. PCPs’ offices do not have the same diagnostic and therapeutic capabilities as EDs. These deficiencies could be addressed through structural changes (e.g., expand capabilities at PCP offices), but the value of this change must be measured against the cost. Alternatively, a better source of illness care may be the ED, as long as the ED structure and processes are optimized to the needs of older patients, such as through geriatric EDs.^9^

Another consideration is developing a more flexible system that can support the wide range of older adults’ needs. Telemedicine is increasingly being used to deliver acute illness care to patients in their homes, making care available when patients want it and without creating other needs, such as transportation to a PCP’s office or an ED. Studies have shown the feasibility, acceptability, and effectiveness of telemedicine to provide acute illness care.^26^,^27^

In the era of on-demand transportation such as Uber, the transportation barrier described by participants is likely surmountable. Developing a more robust and affordable transport system should be possible to support older adults who require in-person treatment.^28^

Finally, a potential individual-level intervention is decision support for patients. Decision support, such as a nurse help line, could assist older adults in choosing the proper site for illness care, and even address the uncertainty and anxiety issues raised by participants. Bolstering this patient support may allow for a more streamlined and efficient system of acute care, but the accuracy of such a help line will need to be evaluated. Help lines have been successfully used for children, but may be inaccurate among complex geriatric patients.^29^

## LIMITATIONS

This study has a few limitations that must be considered. As the study was conducted in a single ED in one mid-sized city, the findings may not be generalizable to different patient populations; the hypotheses generated from this study must be confirmed through larger survey studies. Another limitation is not enrolling patients who obtained acute illness care from their PCPs. However, our goal was to broadly understand the decision-making process of patients who chose to go to the ED for care over their PCPs. Our findings can now build to a future study that compares patients who receive acute illness care in EDs to those who receive care in PCP offices. A final limitation relates to internal validity. Because this is a hypothesis-generating qualitative study with a small number of participants, patients with every presenting condition were not included. Because we did not survey indidivuals who refused to consent to participate in this study, differences may have existed between those who participated and those who refused to participate. Because the interviews occurred after the participant decided to present to the ED for care, we cannot know if the patients’ opinions changed as a result of the experiences in the ED. Thus, these findings must be considered with the caveat that a confirmatory study must be performed.

## CONCLUSION

Older adults integrate a number of factors when deciding whether to obtain acute illness care from an ED rather than their PCPs. These factors relate to personal and social considerations, practical considerations, and perceptions based on previous experiences. Person-centered interventions at the system and individual level should be considered to optimize the care that community-dwelling older adults receive for their acute illnesses.

## Figures and Tables

**Table 1:** Demographic characteristics of participants.

Pseudonym	Gender	Race	Chief complaint
Mandy	Female	White	Knee pain
Joe	Male	White	Unable to urinate
April	Female	White	Fall
May	Female	Black	Motor vehicle crash
June	Female	White	Syncope
Carol	Female	White	Constipation
Mark	Male	White	Bee sting
Peter	Male	Black	Bee sting
David	Male	White	Knee pain
Audrey	Female	White	Hand injury
Ray	Male	White	Arm injury
Mildred	Female	Black	Hand injury
Jenny	Female	White	Abdominal pain/difficulty sleeping
Arthur	Male	White	Syncope
Quinton	Male	White	Abdominal pain

**Table 2:** Representative participant quotations.

	Line	Quote
Limited availability of PCP-based illness care	1	April: “If I need him, I call him, but there’s not too often that he’ll say he wouldn’t take me; he usually always takes me.”
	2	Mark: “I called him and he said…he’ll make time for me and he said I could come right in…they’re very accommodating.”
	3	Peter: “Well, 5:00 in the afternoon the doctors’ offices are closed…I went out here to Urgent Care and they wouldn’t deal with me. They said they would call the ambulance…”
	4	Carol: “…when we had an emergency situation like this, they [the PCP’s office] didn’t respond, which is no good.”
	5	David: “I was amazed that from the time I went through the door to the time I actually had some care, probably 8 minutes, 7 or 8 minutes…(regarding ED care).”
Variable interactions with healthcare providers and systems	6	Joe: “He [PCP] doesn’t spend a lot of time with me, but he seems to listen to what I have to say…I trust him.”
	7	April: “Yeah, he’s a good doctor, I think the world of him…I have a lot of faith in him, I trust him and I think you need that more than anything.”
	8	Carol: “We moved from the Adirondacks and I had to find a doctor and I went through four different doctors before I found a doctor that I could talk to…what decided me [was] not only his efficiency but his caring…”
	9	Mark: “I think for the most part our visits to [the emergency department] have always gone very well for us…”
	10	Mandy: “I have to say I was pleasantly surprised when I came to the ER because I’d heard horror stories about coming here to the ER and when I got here they couldn’t have been more helpful…”
	11	Mandy: “…I tried to talk the doctor into sending me [to his office] that day…He said, ‘I want you in the hospital now.”
	12	Audrey: “We’ve done it in the past through going to the emergency room…[my PCP] wouldn’t want me to come to the office, I knew that.”
	13	June: “[The PCP] sent me straight to the emergency room.”
	14	David: “Well, I can honestly tell you that if I had health-related problems, well like this for instance, this [the ED] is the place I would rather be... I was amazed that from the time I went through the door to the time I actually had some care, probably 8 minutes, 7 or 8 minutes, I was amazed.”
Availability of transportation for illness	15	June’s caregiver: “She does not drive anymore, so obviously all the driving has to come from somebody else…”
	16	May: “Sometimes [the medical cab] don’t come…I’ve missed about four or five appointments messing with them.”
	17	June: “Medicaid has to provide transportation and I have had one of the biggest struggles of anything with [that] transportation system.”
	18	June: “I said ‘well, I’ll have to find somebody to give me a ride and it will take a couple of hours’ and she said ‘if you can’t find someone, then call the ambulance and get in [to the ED].”
	19	Mandy: “[T]hat was the hardest lesson…I had to ask friends. Even though I had helped them a thousand times, it was different when you have to ask, do the asking. You really take, it’s a blow to your self-esteem, you know, who’ve you been all these years, so it is hard.”
Desire to avoid burdening friends and family	20	June: “I have a real need for independence…I have to learn how to let some of that go and accept that I need help from other people and that is a real challenge for me…”
	21	April: “It’s so hard to even ask my own kids.”
	22	Peter: “I don’t burden my people down with my problems, because everybody’s got problems.”
Previous experiences with illnesses	23	Carol: “I got up and thought ‘Oh God, it’s the same situation’…I suspected it had something to do with my bowel…we’ve got to find out what the problem is.”
	24	Mandy: “I guess that’s my personality, to be more proactive…I don’t like to be confused. I’m very unconfident if I don’t understand what is going on around me and I didn’t understand a lot of times.”
	25	June: “If I hadn’t come in [to the ED] I would have always wondered if I should have.”
	26	Audrey: “I am worried about the future, right now we’ve taken care of things the way we know we had to, and if they go and change it on us then I’m going to probably have some problems, I don’t know.”
	27	April: “I was scared. I’m not chicken or anything but I woke up and couldn’t breathe… I did think ‘I can’t die here alone’ and then the more I thought about it the more anxiety and then it was worse…”
	28	Audrey: “We’ve done it in the past through going to the emergency room…[my PCP] wouldn’t want me to come to the office, I knew that.”
	29	Jenny: “My daughter said that if I had any complaints I should come.”
	30	Mark: “She goes ‘you want to go to the emergency [room]...that’s where you’re going and I’ll be there in a few minutes.’ She drove over here and picked me up...and we went.”
	31	April: “And the minute she walked in, I knew what she was going to do...she was right on that phone for 911.”

*PCP*, primary care physician.
